# Supplementary material for: Splice-Junction-Based Mapping of Alternative Isoforms in the Human Proteome
Source: Cell Rep. Author manuscript; Available in PMC 2020 Jan 15. (PMC6961840; doi:10.1016/j.celrep.2019.11.026)
Supplement: 2 [file NIHMS1546469-supplement-2.zip › DF1/PXD010154/AdrenalGland/AdrenalGland_18_CD163_NVIGSLDAYNGQEEDSGSADFR.pdf]

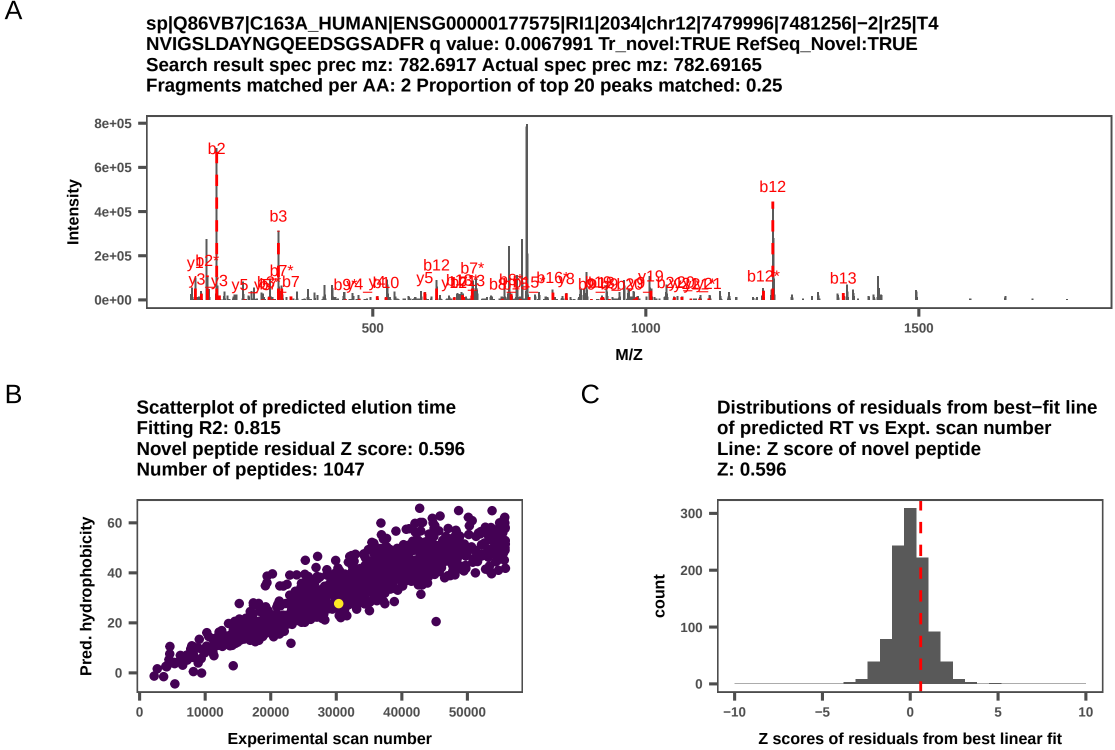
A
sp|Q86VB7|C163A_HUMAN|ENSG00000177575|RI1|2034|chr12|7479996|7481256|−2|r25|T4
NVIGSLDAYNGQEEDSGSADFR q value: 0.0067991 Tr_novel:TRUE RefSeq_Novel:TRUE
Search result spec prec mz: 782.6917 Actual spec prec mz: 782.69165
Fragments matched per AA: 2 Proportion of top 20 peaks matched: 0.25
Intensity
8e+05
6e+05
4e+05
2e+05
0e+00
b2
b3
b12
b12*
b13
b7*
b7
y5
y19
M/Z
500
1000
1500
B
Scatterplot of predicted elution time
Fitting R2: 0.815
Novel peptide residual Z score: 0.596
Number of peptides: 1047
Pred. hydrophobicity
60
40
20
0
0
10000
20000
30000
40000
50000
Experimental scan number
C
Distributions of residuals from best−fit line
of predicted RT vs Expt. scan number
Line: Z score of novel peptide
Z: 0.596
count
300
200
100
0
−10
−5
0
5
10
Z scores of residuals from best linear fit
